# Safety and efficacy of anagrelide in Japanese post-marketing surveillance, with subgroup analyses on the effect of previous cytoreductive therapies, age, and starting dose

**DOI:** 10.1007/s12185-022-03380-2

**Published:** 2022-05-27

**Authors:** Norio Komatsu, Yoshinori Hashimoto, Terumi Baba, Manami Otsuka, Takafumi Akimoto, Jovelle Fernandez

**Affiliations:** 1 Department of Hematology, Juntendo University Graduate School of Medicine, 2-1-1 Hongo, Bunkyo-ku, Tokyo, 113-8421 Japan; 2 Laboratory for the Development of Therapies Against MPN, Juntendo University Graduate School of Medicine, 2-1-1 Hongo, Bunkyo-ku, 113-8421 Tokyo, Japan; 3 Department of Advanced Hematology, Juntendo University Graduate School of Medicine, 2-1-1 Hongo, Bunkyo-ku, Tokyo, 113-8421 Japan; 4 Department of Hematology, Tottori Prefectural Central Hospital, 730 ezu, Tottori City, Tottori 680-0901 Japan; 5 Japan Medical Office, Japan Pharma Business Unit, Takeda Pharmaceutical Company Limited, 1-1, Nihonbashi-Honcho 2-chome, Chuo-ku, Tokyo, 680-0901 Japan

**Keywords:** Anagrelide, Essential thrombocythemia, Japanese, Post-marketing surveillance

## Abstract

**Background:**

In Japan, anagrelide has been approved for use in patients with essential thrombocythemia. Here, the safety and efficacy of anagrelide was assessed in clinical practice as post-marketing surveillance. Subgroup analyses were conducted to compare patients (1) with or without a history of cytoreductive therapy (CRT), (2) <60 or ≥60 years of age, and (3) with an anagrelide starting dose of ≤0.5 mg/day or 1.0 mg/day.

**Methods:**

Data were collected for all patients who received anagrelide, with an observation period of 12 months after treatment initiation.

**Results:**

Of the 648 patients, 54.3% experienced adverse drug reactions (ADRs). The most commonly reported ADRs were headaches, palpitations, and anemia. No significant difference was observed in overall ADRs across patient subgroups. A significantly higher incidence of headaches was observed in patients < 60 years versus those ≥ 60 years (*P* < 0.001). The incidence of anemia and serious ADRs were significantly higher in patients ≥ 60 years, and those with a history of CRT (*P* < 0.05). The discontinuation rate at 6 months was significantly lower in patients started at the lower anagrelide dose (*P* < 0.05). Platelet counts decreased in all analyzed groups.

**Conclusions:**

This surveillance showed that anagrelide has a tolerable safety and efficacy profile.

**Supplementary Information:**

The online version contains supplementary material available at 10.1007/s12185-022-03380-2.

## Introduction

Essential thrombocythemia (ET) is a myeloproliferative neoplasm resulting from driver gene mutations in *JAK2*, *CALR*, and *MPL* in hematopoietic stem cells. It is characterized by increased production of megakaryocytes and platelets [1]. ET is associated with an increased risk of thrombosis and/or hemorrhage as well as progression to myelofibrosis or leukemia which are in turn associated with increased mortality and morbidity [2, 3]. One of the main treatment goals for ET is to reduce the platelet count to prevent the onset of thrombohemorrhagic events [4, 5].

In patients who have a high-risk of developing thrombosis, cytoreductive therapy (CRT) and antiplatelet therapy have been endorsed within some guidelines [6–8]. Anagrelide is a cytoreductive agent, and although its mechanism of platelet reduction is not well understood, it has been theorized to selectively suppress the mRNA expression of GATA‑1 and FOG‑1 transcription factors. This results in the inhibition of megakaryocyte differentiation from hematopoietic stem cells [9]. In addition, anagrelide also suppresses proplatelet formation, thereby reducing platelet counts [10].

A Phase III study in high-risk Japanese patients with ET showed that anagrelide reduced platelet counts with a safety profile consistent with the findings that supported the approval of anagrelide in Europe and the United States [11]. As a result of these findings, anagrelide was granted approval in September 2014 for the treatment of ET in Japan [11, 12]. Only a limited number of patients (*N* = 53) were enrolled in the Phase III Japanese study, which focused on high-risk patients with ET who were refractory or intolerant to hydroxycarbamide [11]. Anagrelide has also been approved for treatment-naïve patients with ET based on the results of two global clinical studies; ANAHYDRET and a Phase IIIb clinical study for anagrelide [13, 14]. Therefore, the safety and efficacy findings for anagrelide in CRT-naïve Japanese patients are limited [15, 16].

Interestingly, there has been less concern regarding secondary malignancy developing in patients taking anagrelide than some alternative therapies, and it has been indicated that a higher proportion of younger patients receive anagrelide [17]. To date, limited data have been presented comparing the safety and efficacy of anagrelide between patients who are < 60 years of age and those who are ≥ 60 years of age. Furthermore, the starting dose of anagrelide, as per the prescribing information, is 1.0 mg/day; however, in the previous Japanese study, the rate of adverse drug reactions (ADRs) was significantly lower in patients taking a lower starting dose of 0.5 mg/day [16].

The aim of this multicenter, post-marketing surveillance was to investigate the safety and efficacy of anagrelide treatment under daily clinical conditions after its approval and introduction in Japan. This surveillance also compared the safety and efficacy of anagrelide in patients: 1) with and without a history of CRT, 2) < 60 and ≥ 60 years of age, and 3) taking different starting doses of anagrelide (≤ 0.5 and 1.0 mg/day).

## Materials and methods

### Surveillance design

This was a non-interventional (observational) post-approval commitment surveillance (clinicaltrials.gov ID: NCT03625895) conducted in Japan to investigate the safety and efficacy of anagrelide in patients with ET. This surveillance was conducted in compliance with the Japanese regulatory requirements of the Good Post-marketing Study Practice. Approval from each institutional ethics committee and written informed consent from the patients was not mandatory. However, any investigation related to the surveillance was performed after the completion of the contracts between each medical institution and Takeda/Shire Pharmaceutical Co. Ltd.

The observational period was 12 months, starting from the day of treatment initiation with anagrelide. Case report forms (CRFs) were collected in two batches during the observational period; CRF Volume 1 was collected from treatment initiation to 6 months and CRF Volume 2 was collected from months 7–12. For the patients who discontinued anagrelide, the data were collected up to the time of discontinuation.

### Patients

Patients who had started treatment with anagrelide between 25 November 2014 and 31 May 2015 in Japan were included in this surveillance. The patient disposition is shown in Supplementary Fig. 4. Patients who had received at least 1 dose of anagrelide after enrollment and had safety data, were included in the safety analysis set. Of the patients in the safety analysis set, those who had their platelet count measured at enrollment and at least once after the start of anagrelide treatment were included in the efficacy analysis set. Because the objective of this surveillance was to investigate the safety and efficacy of anagrelide under daily clinical conditions, ET diagnosis was performed at the physicians’ discretion. Gene mutation analysis and bone marrow biopsies were not mandatory.

The safety and efficacy of anagrelide was compared in patients categorized into the following subgroups: (1) with and without a history of CRT, (2) < 60 and ≥ 60 years of age, and (3) taking different starting doses of anagrelide (≤ 0.5 and 1.0 mg/day). Patients who had received anagrelide before registration of this surveillance were excluded from the subgroup analyses due to the patients’ characteristics at the start of this surveillance being different from those at the time they started treatment with anagrelide.

### Assessments

Treatment-emergent adverse events (TEAEs) considered to be related to anagrelide were defined as ADRs. The ADRs were coded using the Medical Dictionary for Regulatory Activities/J (MedDRA/J), version 22.0. Patients with multiple events of the same pre-defined event category were only recorded once.

The primary efficacy outcome of the surveillance was to evaluate the proportion of patients who had a response in platelet count (< 600 × 10^9^/L) beyond 3 months (≥ 91 days) after the start of treatment with anagrelide. The secondary efficacy outcomes were to evaluate the proportion of patients who: (a) had a platelet response rate < 400 × 10^9^/L beyond 3 months (≥ 91 days) after the start of administration, and (b) achieved a 50% reduction in platelet count at any time after anagrelide administration versus their baseline values.

### Statistical analysis

Patient characteristics, incidence of ADRs, rate of discontinuation, and platelet reduction and control rate were analyzed with the Chi-square or Fisher’s exact test to determine statistical significance. Univariable and multivariable analyses using logistic regression were conducted to identify risk factors for anemia. For these tests, the two-sided significance level of 95% was selected. Factors were considered to be statistically significant when the *P* value was < 0.05. Statistical analyses were performed using Statistical Analysis Software (SAS) Version 9.4 (or higher) within a controlled environment.

## Results

### Patient disposition, baseline demographics, and disease characteristics

Overall, 689 patients were registered in this surveillance (Supplementary Fig. 4). The first group of CRF (Volume 1; data from treatment initiation to 6 months) was collected from 679 patients, and CRF Volume 2 (data from months 7–12) was collected from 458 patients. A total of 648 patients received 1 or more doses of anagrelide and had one or more post-baseline safety assessment (the Safety Analysis set). The Efficacy Analysis set comprised 627 patients.

Of the 648 patients in the safety population, 41.4% (268 patients) were male, and 58.6% (380 patients) were female (Supplementary Table 4). The median age of these patients was 68 years (range 12–99 years), with 61.3% of the patients ≥ 65 years of age. At registration, the median disease duration was 4.5 years and the median platelet count was 888 × 10^9^/L (range 160–3050 × 10^9^/L). An analysis for *JAK2*V617F mutation was performed in 319 patients, of whom 172 patients (53.9%) were positive. A history of thrombohemorrhagic events was documented in 9.9% (64 patients).

### Safety and efficacy across all patients

Of 648 patients in the safety analysis set, ADRs were reported in 54.3% (352 patients) (Supplementary Table 5). The most frequently reported ADRs (as per MedDRA/J, version 22.0. terms) were headache (15.4%; 100 patients), palpitations (13.9%; 90 patients), anemia (6.6%; 43 patients), diarrhea (5.9%; 38 patients), and peripheral edema (3.5%; 23 patients). The incidence of serious ADRs was reported in 8.2% (53 patients [Supplementary Table 5]). The most frequently reported serious ADRs were cardiac failure (0.9%; 6 patients), followed by atrial fibrillation, cerebral infarction, electrocardiogram QT prolongation, and renal impairment (0.5%; 3 patients each, respectively). Serious ADRs with an outcome of death included: cardiac failure, cerebral infarction, and cerebral vascular stenosis, reported in 1 patient each (0.2%). Most of the ADRs occurred within 6 months of starting treatment with anagrelide (322/352 patients). During the surveillance period, 38.1% (247 patients) in the safety analysis set discontinued treatment with anagrelide. Of these patients, 194 discontinued treatment within the first 6 months. Although multiple reasons for the discontinuation of treatment with anagrelide were recorded, the most common reason was adverse events, as reported for 117 patients.

The primary efficacy outcome result (platelet count < 600 × 10^9^/L beyond 3 months [≥ 91 days]) was 64.7% (282/436 patients). For the secondary efficacy outcomes, the proportion of patients whose platelet count decreased below 400 × 10^9^/L beyond 3 months (≥ 91 days) and by 50% from baseline was 33.0% (175/531 patients) and 40.2% (251/624 patients), respectively (Supplementary Fig. 5).

### Safety and efficacy according to patients’ history of CRT

A subgroup analysis was conducted according to the patients’ history of CRT. Group A comprised 146 patients who had no history of CRT (CRT naïve), and Group B comprised 478 patients who had a history of CRT (not-CRT naïve). Patient characteristics according to their history of CRT are shown in Supplementary Table 6. The patients in Group B were significantly older and were more likely to have had a history of thrombohemorrhagic events compared with Group A (*P* < 0.001 and *P* < 0.05, respectively). As a result, there were significantly more high-risk patients in Group B than in Group A (*P* < 0.001).

The incidence of all ADRs was 49.3% (72 patients) in Group A and 56.7% (271 patients) in Group B (Table 1). Although the rate of ADRs was slightly lower in Group A than in Group B, the difference was not significant (*P* = 0.129). Notably, the incidence of anemia was significantly higher in Group B than in Group A (reported in 37 [7.7%] and 4 patients [2.7%], respectively; *P* < 0.05). Of the serious ADRs, 3.4% (5 patients) were reported in Group A and 9.6% (46 patients) were reported in Group B (*P* < 0.05). The most frequently reported serious ADR across both groups was cardiac failure (1.0%; 6 patients); however, this ADR was observed only in Group B.

**Table 1:** Incidence of ADRs according to patients’ history of CRT, as per MedDRA, version 22.0. terms

ADRs, *n* (%)	Total (*N* = 624)	Group A CRT naïve (*n* = 146)	Group B Not-CRT naïve (*n* = 478)	Group A vs Group B *P* value
All ADRs	343 (55.0)	72 (49.3)	271 (56.7)	0.129
Headache	99 (15.9)	26 (17.8)	73 (15.3)	0.518
Palpitations	90 (14.4)	19 (13.0)	71 (14.9)	0.687
Anemia	41 (6.6)	4 (2.7)	37 (7.7)	< 0.05
Diarrhea	37 (5.9)	7 (4.8)	30 (6.3)	0.689
Peripheral edema	23 (3.7)	5 (3.4)	18 (3.8)	1.000
Other	231 (37.0)	44 (30.1)	187 (39.1)	0.051
All serious ADRs	51 (8.2)	5 (3.4)	46 (9.6)	< 0.05
Cardiac failure	6 (1.0)	0 (0.0)	6 (1.3)	NA
Atrial fibrillation	3 (0.5)	0 (0.0)	3 (0.6)	NA
Cerebral infarction	3 (0.5)	0 (0.0)	3 (0.6)	NA
Electrocardiogram QT prolonged	3 (0.5)	1 (0.7)	2 (0.4)	0.551
Renal impairment	3 (0.5)	0 (0.0)	3 (0.6)	NA
Other	36 (5.8)	4 (2.7)	32 (6.7)	0.102

*ADRs* adverse drug reactions, *CRT* cytoreductive therapy, *NA* not applicable

**Fig. 1:**
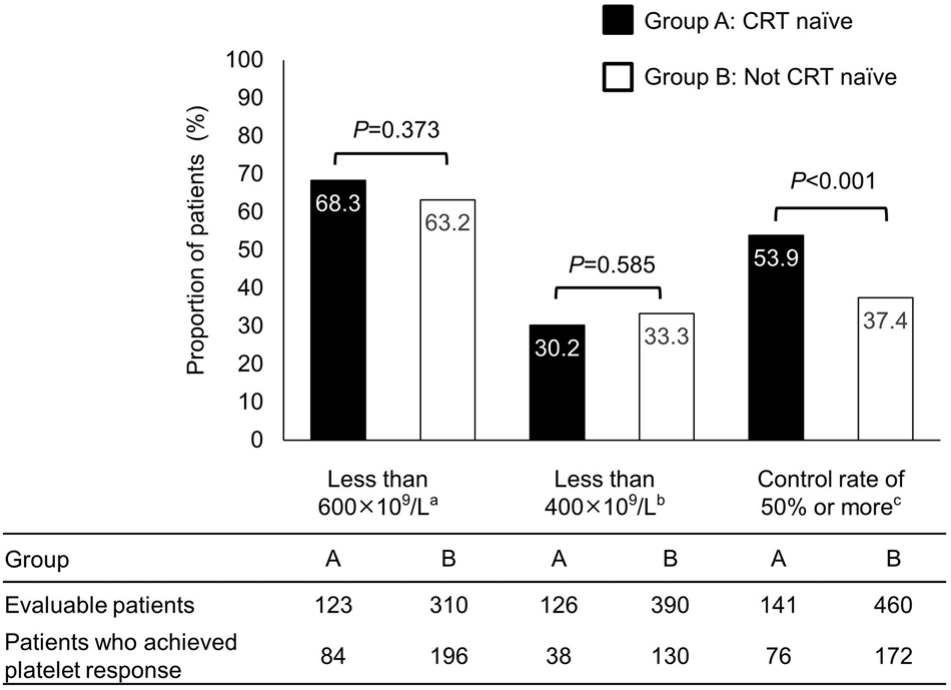
Platelet reduction and control rate for patients in the Efficacy Analysis Set, comparing those who were CRT naïve (Group A) and those were not-CRT naive (Group B). ^a^Platelet count had decreased below 600 × 10^9^/L beyond 3 months (≥ 91 days) after baseline. ^b^Platelet count had decreased below 400×10^9^/L beyond 3 months (≥ 91 days) after baseline. ^c^Platelet count had decreased by 50% or more at any time after anagrelide administration from that at baseline. *CRT* cytoreductive therapy

The proportion of patients whose platelet count had decreased by 50% or more from baseline was 53.9% in Group A, and this was significantly higher than that reported for Group B (*P* < 0.001, Fig. 1). Conversely, there were no significant differences in the proportion of patients whose platelet count had decreased below 600 × 10^9^/L or 400 × 10^9^/L beyond 3 months (≥ 91 days) after baseline between Group A and Group B (*P* = 0.373, and *P* = 0.585, respectively).

### Safety and efficacy according to patients’ age

The safety and efficacy findings were compared between patients < 60 years of age (Group C), and patients ≥ 60 years of age (Group D). One patient was excluded, because their age was unknown. The number of patients with a history of CRT was significantly higher in Group D than in Group C, and platelet count was significantly lower in Group D than in Group C (*P* < 0.001 and *P* < 0.05, respectively, Supplementary Table 7). Furthermore, there were more patients with a history of complications, including cardiac disorders in Group D than in Group C (*P* < 0.001).

The incidence of all ADRs was 53.9% (97/180 patients) in Group C and 55.5% (246/443 patients) in Group D, and there was no significant difference between the groups (*P* = 0.723, Table 2). Interestingly, headache was reported for 27.8% (50 patients) of Group C, and this incidence was significantly higher than that reported for Group D (11.1%, 49 patients; *P* < 0.001). In contrast, anemia was reported for 8.1% (36 patients) in Group D and 2.8% in Group C (5 patients; *P* < 0.05). Similarly, the majority of patients who experienced serious ADRs were in Group D (*P* < 0.001).

**Table 2:** Incidence of ADRs according to patients’ age as per MedDRA, version 22.0. terms

ADRs, *n* (%)	Total (*N* = 623)	Group C < 60 years (*n* = 180)	Group D ≥ 60 years (*n* = 443)	Group C vs Group D *P* value
All ADRs	343 (55.1)	97 (53.9)	246 (55.5)	0.723
Headache	99 (15.9)	50 (27.8)	49 (11.1)	< 0.001
Palpitations	90 (14.4)	31 (17.2)	59 (13.3)	0.211
Anemia	41 (6.6)	5 (2.8)	36 (8.1)	< 0.05
Diarrhea	37 (5.9)	8 (4.4)	29 (6.5)	0.356
Peripheral edema	23 (3.7)	6 (3.3)	17 (3.8)	1.000
Other	231 (37.1)	57 (31.7)	174 (39.3)	0.082
All serious ADRs	51 (8.2)	3 (1.7)	48 (10.8)	< 0.001
Cardiac failure	6 (1.0)	1 (0.6)	5 (1.1)	0.678
Atrial fibrillation	3 (0.5)	0 (0.0)	3 (0.7)	NA
Cerebral infarction	3 (0.5)	0 (0.0)	3 (0.7)	NA
Electrocardiogram QT prolonged	3 (0.5)	0 (0.0)	3 (0.7)	NA
Renal impairment	3 (0.5)	0 (0.0)	3 (0.7)	NA
Other	36 (5.8)	2 (1.1)	34 (7.7)	< 0.001

*ADRs* adverse drug reactions, *NA* not applicable

**Fig. 2:**
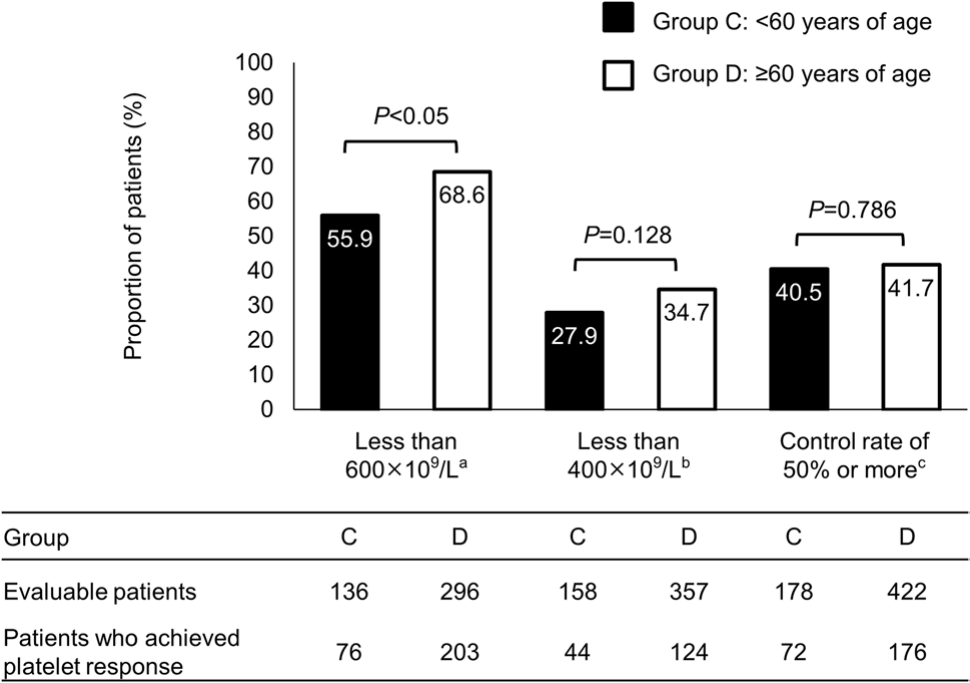
Platelet reduction and control rate for patients in the efficacy analysis set, comparing those who were < 60 years of age (Group C) and those who were ≥ 60 years of age (Group D). ^a^Platelet count had decreased below 600 × 10^9^/L beyond 3 months (≥ 91 days) after baseline. ^b^Platelet count had decreased below 400 × 10^9^/L beyond 3 months (≥ 91 days) after baseline. ^c^Platelet count had decreased by 50% or more at any time after anagrelide administration from that at baseline

In the efficacy analysis, the primary outcome (platelet count < 600 × 10^9^/L beyond 3 months [≥ 91 days] after baseline) was achieved for a significantly higher proportion of Group D than of Group C (*P* < 0.05, Fig. 2). No significant differences were observed for the secondary outcomes, including the proportion of patients with a platelet response rate of < 400 × 10^9^/L beyond 3 months (≥ 91 days) after baseline and the proportion of patients who achieved a 50% reduction in platelet count versus their baseline values (*P* = 0.128, and *P* = 0.786, respectively, Fig. 2).

### Safety and efficacy according to the starting dose of anagrelide

The number of patients who started on ≤ 0.5 mg/day of anagrelide was 135 (Group E), and 475 patients started on 1.0 mg/day (Group F). The prescribing information specifies that the starting dose of anagrelide is 1.0 mg/day, and consequently, the 14 patients who started with a dose of more than 1.0 mg/day were excluded from this analysis. Although more patients in Group F had cardiac disorders, there were no significant differences between the patient characteristics when comparing Groups E and F (Supplementary Table 8). The incidence of all ADRs was reported for 48.9% (66 patients) of patients in Group E and 56.6% (269 patients) of patients in Group F, respectively (Table 3). There was no significant difference between these groups (*P* = 0.118). Similarly, there was no significant difference between groups when assessing the rates of all serious ADRs (*P* = 0.859). The main serious ADRs were cardiac disorders (such as cardiac failure or atrial fibrillation), most of which occurred in Group F. Furthermore, the discontinuation rate of anagrelide within the first 6 months of treatment was significantly lower in Group E than in Group F (*P* < 0.05, Supplementary Table 9). Among the many reasons for discontinuation, the most common cause was adverse events.

**Table 3:** Incidence of ADRs according to the starting dose of anagrelide as per MedDRA, version 22.0. terms

ADRs, *n* (%)	Total *N* = 610	Group E (≤ 0.5 mg/day starting dose) *n* = 135	Group F (1.0 mg/day starting dose) *n* = 475	Group E vs Group F *P* value
All ADRs	335 (54.9)	66 (48.9)	269 (56.6)	0.118
Headache	95 (15.6)	18 (13.3)	77 (16.2)	0.501
Palpitations	89 (14.6)	16 (11.9)	73 (15.4)	0.337
Anemia	40 (6.6)	6 (4.4)	34 (7.2)	0.327
Diarrhea	37 (6.1)	10 (7.4)	27 (5.7)	0.422
Peripheral edema	23 (3.8)	2 (1.5)	21 (4.4)	0.131
Other	224 (36.7)	47 (34.8)	177 (37.3)	0.615
All serious ADRs	50 (8.2)	10 (7.4)	40 (8.4)	0.859
Cardiac failure	6 (1.0)	0 (0.0)	6 (1.3)	NA
Atrial fibrillation	3 (0.5)	0 (0.0)	3 (0.6)	NA
Cerebral infarction	3 (0.5)	0 (0.0)	3 (0.6)	NA
Electrocardiogram QT prolonged	3 (0.5)	0 (0.0)	3 (0.6)	NA
Renal impairment	3 (0.5)	1 (0.7)	2 (0.4)	0.529
Other	35 (5.7)	9 (6.7)	26 (5.5)	0.675

*ADRs* adverse drug reactions, *NA* not applicable

**Fig. 3:**
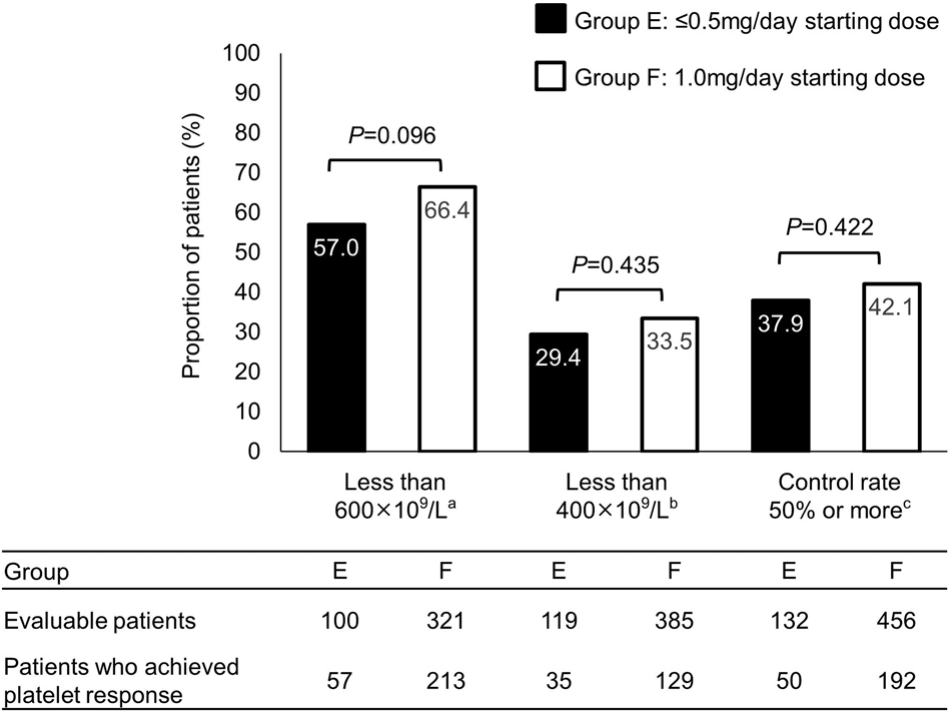
Platelet reduction and control rate for patients in the Efficacy Analysis Set comparing those who started anagrelide treatment on ≤ 0.5 mg/day (Group E) and those who started on 1.0 mg/day (Group F). ^a^Platelet count had decreased below 600 × 10^9^/L beyond 3 months (≥ 91 days) after baseline. ^b^Platelet count had decreased below 400 × 10^9^/L beyond 3 months (≥ 91 days) after baseline. ^c^Platelet count had decreased by 50% or more at any time after anagrelide administration from that at baseline

Although the efficacy findings for Group F (66.4% [213/321 patients]) appeared to be more favorable than those of Group E (57.0% [57/100 patients]), there was no significant difference in the primary outcome of the efficacy analysis (platelet count < 600 × 10^9^/L beyond 3 months [≥ 91 days] after baseline; *P* = 0.096, Fig. 3).

## Discussion

In this surveillance, subgroup analyses findings may assist with the management of anagrelide treatment in certain groups of patients. To the best of our knowledge, this is the first evaluation of patients treated with anagrelide comparing those: (1) with or without a history of CRT; and (2) < 60 and ≥ 60 years of age.

The most frequently reported ADR in the Phase III study investigating anagrelide in Japanese patients was anemia (47.2%, 25/53 patients) [11]. Anemia in both this surveillance and the Phase III study may be due to the myelosuppression resulting from a history of CRT. In this surveillance, the incidence of anemia was significantly higher in Group B, patients who had a history of CRT, and Group D, patients ≥ 60 years of age (Tables 1, 2). However, analyses to identify risk factors for anemia suggested that age ≥ 60 years and history of CRT may have been confounding factors (Supplementary Table 10). Although history of CRT was not an independent risk factor for anemia in the present analysis, patients with history of CRT should be closely monitored for worsening anemia when treated with anagrelide, partly because of myelosuppression caused by previous CRT. Additionally, the incidence of anemia was 6.6% (43/648 patients) in the overall patient population, and this was similar to the rates reported in the UK-PT1 study (7.9%) and the ANAHYDRET study (9.0%) [13, 18]. These results suggest that the incidence of anemia is similar between Japanese and non-Japanese patients; however, hemoglobin levels should still be monitored under daily clinical conditions, especially for patients with a history of CRT, and those who are ≥ 60 years of age.

The most frequent serious ADRs were related to cardiac disorders, which occurred in Group B (history of CRT), Group D (≥ 60 years of age), and Group F (1.0 mg/day starting dose; Tables 1, 2, 3). The patients in these groups either had a history of, or experienced complications of cardiac disorders at baseline (Supplementary Tables 6, 7, and 8). Of all 9 patients with serious ADRs of cardiac failure (*n* = 6) or atrial fibrillation (*n* = 3), 5 patients had a history of cardiac complications. A previous study investigating anagrelide use in 55 patients showed that it was difficult to predict cardiac adverse events, even if in-depth cardiovascular monitoring was performed [19]. Another study showed that the starting dose of anagrelide was significantly higher in patients who experienced cardiovascular adverse events compared with the patients who did not experience any cardiovascular events [20]. These findings indicate that cardiovascular monitoring should be regularly conducted in daily clinical practice to identify and manage any cardiac adverse events in patients taking anagrelide. This is particularly relevant for patients who have a history or have had complications of cardiac disorders prior to the start of treatment. In addition, it may be beneficial to start patients on a lower dose of anagrelide, and then gradually increase the dose while monitoring for any cardiac adverse events [16].

Anagrelide is able to inhibit phosphodiesterase III activity, leading to peripheral vasodilation, which can cause headaches [21]. The cause of patients < 60 years of age having more headaches than those ≥ 60 years of age within this surveillance is unknown. It is worth noting that because headaches are reported to be a symptom of ET, it cannot be ruled out that the headaches that physicians reported as ADRs following treatment with anagrelide could instead have been caused by ET. The median dose of anagrelide was 1.50 mg (range 0.50–7.44 mg) in patients < 60 years of age versus 1.44 mg (range 0.25–5.13 mg) in patients ≥ 60 years of age, and no significant difference was observed (*P* = 0.094). One possible reason for younger patients experiencing more headaches is that their blood vessels have more elasticity and so are more flexible. It is recommended that more attention should be paid to headaches in younger patients who are taking anagrelide and pain relief (e.g., acetaminophen) may be prescribed to assist with this if needed.

As per the efficacy analysis findings, the median daily dose of anagrelide was 1.29 mg (range 0.25–3.57 mg) in Group E (≤ 0.5 mg/day starting dose) and 1.47 mg (range 0.31–7.44 mg) in Group F (1.0 mg/day starting dose), and the difference between the groups was significant (*P* < 0.001). The findings within this surveillance agree with those of a previous study investigating anagrelide starting doses, which found that the lower starting dose of 0.5 mg/day resulted in fewer discontinuations [16]. Interestingly, in terms of the primary outcome, Group F showed more improvement than Group E, and although this difference was not significant and no differences were noted between the two groups for the secondary efficacy outcomes, this result suggests that a lower starting dose should not affect treatment efficacy (Fig. 3). Taken together, these findings suggest that starting patients on a lower dose of anagrelide may reduce the risk of developing adverse events, and in turn, avoid treatment discontinuation due to adverse events.

In the subgroup analysis, significant differences were observed for the secondary outcome (control rate of 50% or more); however, this was not observed in the primary outcome between Group A; CRT-naïve patients and Group B; not-CRT naïve (Fig. 1). The reason for this is likely that Group A patients started anagrelide treatment with a higher platelet count and many patients may therefore have had a platelet count that decreased below 50% or more, but not below < 600 × 10^9^/L. Alternatively, these patients were not targeted below < 600 × 10^9^/L, because Group A had a higher proportion of low-risk patients (Supplementary Table 6).

The primary efficacy outcome according to age subgroups was significantly higher in Group D (patients ≥ 60 years of age) than in Group C (patients < 60 years of age [Fig. 2]). The patient demographic data for the safety analysis set indicated that almost all patients in Group C were low risk (95.0%); however, 113 patients (62.8%) had a history of CRT (Supplementary Table 7). Nevertheless, the platelet count had been significantly higher at baseline in the patients of Group C than those of Group D (*P* < 0.05, Supplementary Table 7), which may explain the above result. Interestingly, there were more *JAK2*V617F negative patients in Group C (33.9%, 61 patients) compared with Group D (18.1%, 80 patients). Overall, there were no significant differences observed across each secondary outcome (Fig. 2), and taken together, these findings indicate that the efficacy of anagrelide should be favorable even for patients without the *JAK2*V617F mutation.

This surveillance has several limitations. Gene mutation analysis and bone marrow biopsies were not performed for some patients. As a result, the patients who could have polycythemia vera or prefibrotic myelofibrosis according to the World Health Organization 2016 criteria, may be included in this analysis [22]. In this surveillance, ADRs were reported at the attending physicians’ discretion, and as a result, may be underestimated. Serious hemorrhage was reported in 6 patients (0.93%), and all cases recovered. Of these, 5 patients were receiving concomitant aspirin treatment. Although anagrelide treatment may raise concerns regarding hemorrhagic risk in combination with antiplatelet therapy (e.g., aspirin), or disease transformation (e.g., myelofibrosis), these conditions were not further investigated in this surveillance. Future studies will need to address these issues in more detail.

In conclusion, the safety and efficacy of anagrelide are supported by the findings in this surveillance, which found anagrelide to be well tolerated under daily clinical conditions. It is recommended that patients taking anagrelide ≥ 60 years of age and those with a history of CRT are monitored for signs of anemia and other serious ADRs. Care should also be taken regarding headaches in patients < 60 years of age. Starting patients on a lower dose of anagrelide therapy may reduce the risk of adverse events and also avoid treatment discontinuation.

## Supplementary Information

Below is the link to the electronic supplementary material.

Supplementary file1 (DOCX 305 KB)

Supplementary file2 (DOCX 48 KB)

## Data Availability

The datasets, including the redacted surveillance protocol, redacted statistical analysis plan, and individual participants’ data supporting the results reported in this article, will be made available within 3 months from initial request to researchers who provide a methodologically sound proposal. The data will be provided after its de-identification, in compliance with applicable privacy laws, data protection, and requirements for consent and anonymization.
