# Supplementary material for: Safety and efficacy of anagrelide in Japanese post-marketing surveillance, with subgroup analyses on the effect of previous cytoreductive therapies, age, and starting dose
Source: Int J Hematol. 2022 May 27;116(4):570–8. doi: 10.1007/s12185-022-03380-2 (PMC9515010; doi:10.1007/s12185-022-03380-2)
Supplement: Supplementary file 1 — Supplementary file1 (DOCX 305 KB) [file 12185_2022_3380_MOESM1_ESM.docx]

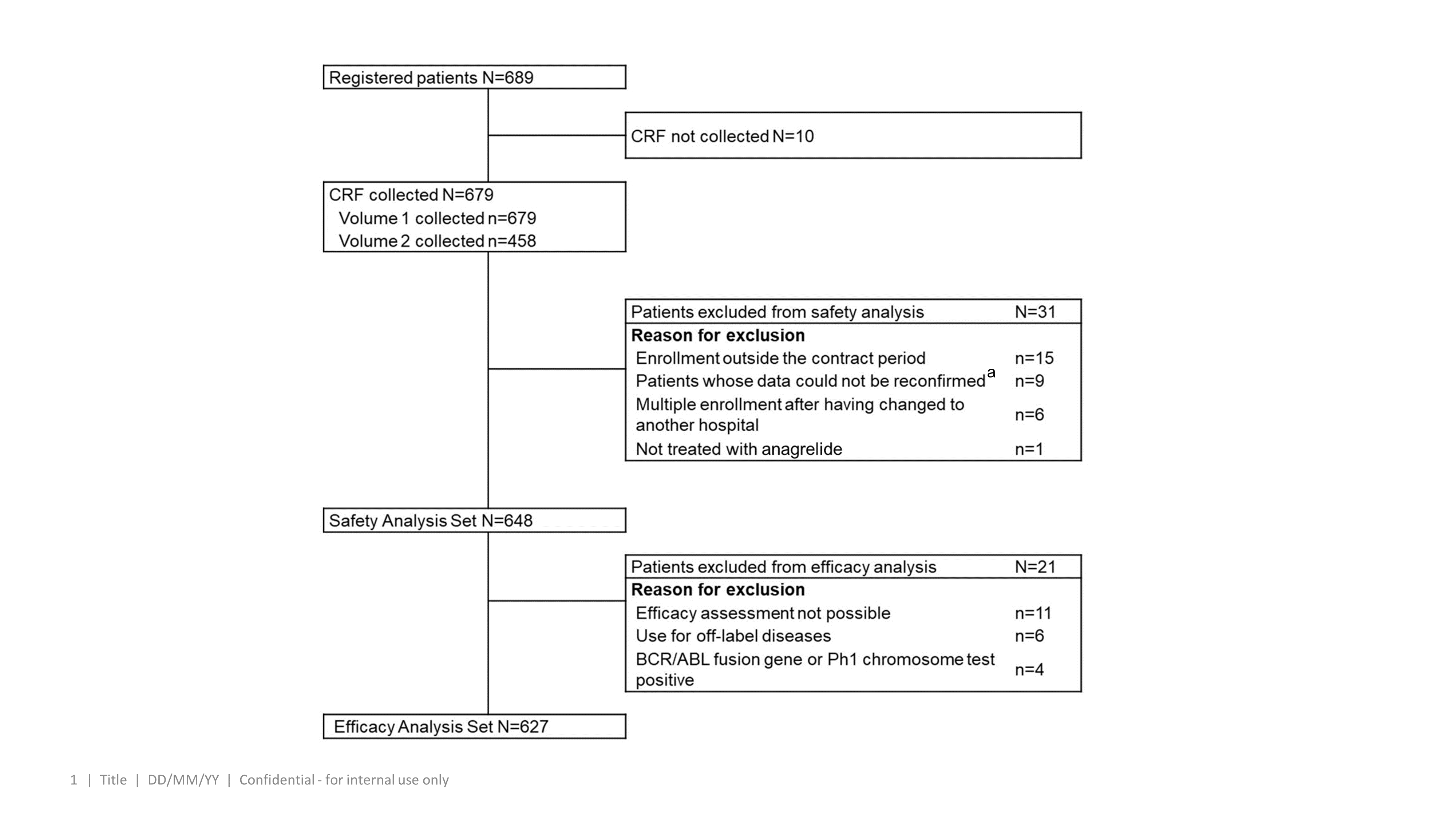


**Supplementary Fig 4** Patient disposition from the time of surveillance registration to the identification of the safety and efficacy analysis populations, detailing reasons for exclusion at each stage

^a^For these patients some of the data appeared to be contradictory, however no resolution was reached. CRF: case report form; Ph1: Philadelphia


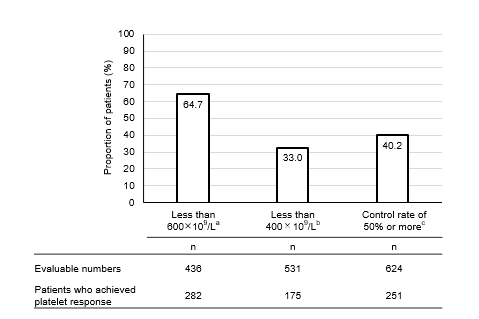


**Supplementary Fig 5** Platelet reduction and control rate for the Efficacy Analysis Set

^a^Platelet count had decreased below 600×10^9^/L beyond 3 months (≥91 days) after baseline

^b^Platelet count had decreased below 400×10^9^/L beyond 3 months (≥91 days) after baseline

^c^Platelet count had decreased by 50% or more at any time after anagrelide administration from that at baseline

**Supplementary Table 4. Patient demographics and disease characteristics at registration: Safety Analysis Set**

| Characteristics | Patients (N=648) |
| --- | --- |
| Sex, n (%) |  |
| Male | 268 (41.4) |
| Female | 380 (58.6) |
| Age median (years), range | 68.0 (12–99) |
| Age category (years), n (%) |  |
| ≤17 | 2 (0.3) |
| 18-64 | 248 (38.3) |
| ≥65 | 397 (61.3) |
| Unknown | 1 (0.2) |
| History of ET (years), median (range) | 4.5 (0–33) |
| Platelet count (×10^9^/L), median (range) | 888 (160–3050) |
| *JAK2*V617F mutation status, n (%) |  |
| Positive | 172 (26.5) |
| Negative | 147 (22.7) |
| Not done | 325 (50.2) |
| Unknown | 4 (0.6) |
| History of thrombo-hemorrhagic events, n (%) | 64 (9.9) |

ET, essential thrombocythemia.

**Supplementary Table 5.** **Adverse drug reactions occurring in ≥2% of patients and major serious adverse reactions as per MedDRA, version 22.0. terms**

| **ADRs, n (%)** | **Patients (N=648)** |
| --- | --- |
| **Overall ADRs** | **352 (54.3)** |
| Headache | 100 (15.4) |
| Palpitations | 90 (13.9) |
| Anemia | 43 (6.6) |
| Diarrhea | 38 (5.9) |
| Peripheral edema | 23 (3.5) |
| Hepatic function abnormal | 17 (2.6) |
| Malaise | 16 (2.5) |
| Edema | 16 (2.5) |
| Dizziness | 13 (2.0) |
| **Serious ADRs** | **53 (8.2)** |
| Cardiac failure | 6 (0.9) |
| Atrial fibrillation | 3 (0.5) |
| Cerebral infarction | 3 (0.5) |
| Electrocardiogram QT prolonged | 3 (0.5) |
| Renal impairment | 3 (0.5) |

ADRs, adverse drug reactions

**Supplementary Table 6. Patient characteristics at registration according to patients’ history of CRT: Safety Analysis Set**

| **Characteristics** | **Total  N=624** | **Group A CRT naïve  n=146** | **Group B Not CRT naïve  n=478** | **Group A  vs Group B *P* value** |
| --- | --- | --- | --- | --- |
| Sex, n (%) |  |  |  | 0.774 |
| Male | 258 (41.3) | 62 (42.5) | 196 (41.0) |  |
| Female | 366 (58.7) | 84 (57.5) | 282 (59.0) |  |
| Age (years), median (range) | 68.0 (12–99) | 62.0 (12–99) | 69.0 (19–93) | <0.001 |
| History of ET (years), median (range) | 4.3 (0–33) | 0.9 (0–25) | 5.4 (0–33) | <0.001 |
| Platelet count (×10^9^/L), median (range) | 901 (160–3050) | 1076 (400–2650) | 839 (160–3050) | <0.001 |
| *JAK2*V617F mutation status, n (%) |  |  |  | NA |
| Positive | 168 (26.9) | 42 (28.8) | 126 (26.4) |  |
| Negative | 142 (22.8) | 36 (24.7) | 106 (22.2) |  |
| Not Done | 312 (50.0) | 67 (45.9) | 245 (51.3) |  |
| Unknown | 2 (0.3) | 1 (0.7) | 1 (0.2) |  |
| Combination with other CRT  during follow-up, n (%) | 277 (44.4) | 0 (0) | 277 (57.9) | <0.001 |
| Risk category^1^, n (%) |  |  |  | <0.001 |
| Low | 171 (27.4) | 65 (44.5) | 106 (22.2) |  |
| High | 453 (72.6) | 81 (55.5) | 372 (77.8) |  |
| Medical history/complications |  |  |  |  |
| Cardiac disorders, n (%) | 66 (10.6) | 7 (4.8) | 59 (12.3) | <0.05 |
| Thrombohemorrhagic events, n (%) | 62 (9.9) | 6 (4.1) | 56 (11.7) | <0.05 |

^1^Low-risk: <60 years of age and no thrombosis history; high-risk: ≥60 years of age or thrombosis history. CRT: cytoreductive therapy; ET: essential thrombocythemia; NA: not applicable.

**Supplementary Table 7. Patient characteristics at registration according to patients' age: Safety Analysis Set**

| **Characteristics** | **Total N=623** | **Group C <60 years n=180** | **Group D ≥60 years n=443** | **Group C  vs Group D *P* value** |
| --- | --- | --- | --- | --- |
| Sex, n (%) |  |  |  | 0.789 |
| Male | 258 (41.4) | 73 (40.6) | 185 (41.8) |  |
| Female | 365 (58.6) | 107 (59.4) | 258 (58.2) |  |
| Age (years), median (range) | 68.0 (12–99) | 46.0 (12–59) | 73.0 (60–99) | <0.001 |
| History of ET (years), median (range) | 4.3 (0–33) | 3.2 (0–23) | 4.6 (0–33) | <0.05 |
| Platelet count (×10^9^/L), median (range) | 899 (160–3050) | 998 (300–2650) | 867 (160–3050) | <0.05 |
| *JAK2*V617F mutation status, n (%) |  |  |  | NA |
| Positive | 168 (27.0) | 47 (26.1) | 121 (27.3) |  |
| Negative | 141 (22.6) | 61 (33.9) | 80 (18.1) |  |
| Not Done | 312 (50.1) | 71 (39.4) | 241 (54.4) |  |
| Unknown | 2 (0.3) | 1 (0.6) | 1 (0.2) |  |
| History of CRT, n (%) | 478 (76.7) | 113 (62.8) | 365 (82.4) | <0.001 |
| Combination with other CRT  during follow-up, n (%) | 277 (44.5) | 72 (40.0) | 205 (46.3) | 0.156 |
| Risk category^1^, n (%) |  |  |  | <0.001 |
| Low | 171 (27.4) | 171 (95.0) | 0 (0.0) |  |
| High | 452 (72.6) | 9 (5.0) | 443 (100.0) |  |
| Medical history/complications |  |  |  |  |
| Cardiac disorders, n (%) | 66 (10.6) | 5 (2.8) | 61 (13.8) | <0.001 |
| Thrombohemorrhagic events, n (%) | 62 (10.0) | 9 (5.0) | 53 (12.0) | <0.05 |

^1^Low-risk: <60 years of age and no thrombosis history; high-risk: ≥60 years of age or thrombosis history. CRT: cytoreductive therapy; ET: essential thrombocythemia; NA: not applicable.

**Supplementary Table 8. Patient characteristics at baseline according to the starting dose of anagrelide: Safety Analysis Set**

| **Characteristics** | **Total N=610** | **Group E ≤0.5mg/day starting dose  n=135** | **Group F 1.0mg/day starting dose n=475** | **Group E  vs Group F *P* value** |
| --- | --- | --- | --- | --- |
| Sex, n (%) |  |  |  | 0.692 |
| Male | 251 (41.1) | 58 (43.0) | 193 (40.6) |  |
| Female | 359 (58.9) | 77 (57.0) | 282 (59.4) |  |
| Age (years), median (range) | 68.0 (12–99) | 67.0 (12–99) | 68.0 (19–93) | 0.617 |
| History of ET (years), median (range) | 4.5 (0–33) | 3.9 (0–22) | 4.4 (0–33) | 0.562 |
| Platelet count (×10^9^/L), median (range) | 888 (160–3050) | 932 (300–2650) | 896 (160–3050) | 0.999 |
| *JAK2*V617F mutation status, n (%) |  |  |  | NA |
| Positive | 163 (26.7) | 47 (34.8) | 116 (24.4) |  |
| Negative | 140 (23.0) | 37 (27.4) | 103 (21.7) |  |
| Not Done | 305 (50.0) | 51 (37.8) | 254 (53.5) |  |
| Unknown | 2 (0.3) | 0 (0.0) | 2 (0.4) |  |
| History of CRT, n (%) | 469 (76.9) | 103 (76.3) | 366 (77.1) | 0.908 |
| Combination with other CRT  during follow-up, n (%) | 275 (45.1) | 64 (47.4) | 211 (44.4) | 0.558 |
| Risk category^1^, n (%) |  |  |  | 1.000 |
| Low | 164 (26.9) | 36 (26.7) | 128 (26.9) |  |
| High | 446 (73.1) | 99 (73.3) | 347 (73.1) |  |
| Medical history/complications |  |  |  |  |
| Cardiac disorders, n (%) | 64 (10.5) | 8 (5.9) | 56 (11.8) | 0.056 |
| Thrombohemorrhagic events, n (%) | 60 (9.8) | 18 (13.3) | 42 (8.8) | 0.140 |

^1^Low-risk: <60 years of age and no thrombosis history; high-risk: ≥60 years of age or thrombosis history. CRT: cytoreductive therapy; ET: essential thrombocythemia; NA: not applicable.

**Supplementary Table 9. Rate of discontinuation from anagrelide along with the reason for discontinuation according to the anagrelide starting dose:
Safety Analysis Set**

1. **From treatment initiation to 6 months**

|  | | | **Group E  (≤0.5mg/day  starting dose)** | | **Group F  (1.0mg/day  starting dose)** | **Group E  vs Group F *P* value** | |
| --- | --- | --- | --- | --- | --- | --- | --- |
| Safety Analysis Set, n (%) | | | | 135 (100.0) | 475 (100.0) | | NA |
| Subjects discontinued, n (%) | | | | 30 (22.2) | 157 (33.1) | | <0.05 |
|  | | Reason for withdrawal (multiple), n | | | | | |
|  | |  | Adverse event | 13  10  1  3  5  0  1 | 85  31  3  18  5  12  15 | |  |
|  | |  | Withdrawal by patient |  |  |  |  |
|  | |  | Lost to follow up |  |  |  |  |
|  | |  | Lack of efficacy |  |  |  |  |
|  | |  | Changing hospital |  |  |  |  |
|  | |  | Patient death |  |  |  |  |
|  | |  | Other |  |  |  |  |

1. **From 7 to 12 months**

|  | | | **Group E  (≤0.5mg/day  starting dose)** | **Group F  (1.0mg/day  starting dose)** | | **Group E  vs Group F *P* value** | |
| --- | --- | --- | --- | --- | --- | --- | --- |
| Safety Analysis Set, n (%) | | | 102 (100.0) | 306 (100.0) | | | NA |
| Subjects discontinued, n (%) | | | 14 (13.7) | 36 (11.8) | | | 0.603 |
|  | Reason for withdrawal (multiple), n | | |  |  | |  |
|  |  | Adverse event | 6  3  1  1  0  3  0 | 11  3  1  5  6  6  6 | | |  |
|  |  | Withdrawal by patient |  |  |  |  |  |
|  |  | Lost to follow up |  |  |  |  |  |
|  |  | Lack of efficacy |  |  |  |  |  |
|  |  | Changing hospital |  |  |  |  |  |
|  |  | Patient death |  |  |  |  |  |
|  |  | Other |  |  |  |  |  |

NA: not applicable.
