# Supplementary material for: Safety and efficacy of anagrelide in Japanese post-marketing surveillance, with subgroup analyses on the effect of previous cytoreductive therapies, age, and starting dose
Source: Int J Hematol. 2022 May 27;116(4):570–8. doi: 10.1007/s12185-022-03380-2 (PMC9515010; doi:10.1007/s12185-022-03380-2)
Supplement: Supplementary file 2 — Supplementary file2 (DOCX 48 KB) [file 12185_2022_3380_MOESM2_ESM.docx]

**Supplementary Table 10. Univariable and multivariable analyses to identify risk factors for anemia: Safety Analysis Set**

| Variables | Univariable | | | |  | Multivariable | | | | |
| --- | --- | --- | --- | --- | --- | --- | --- | --- | --- | --- |
|  | Point Estimate | 95%CI | | *p* value |  | Point  Estimate | 95%CI | | *p* value | |
| Age ≥60 years | 3.101 | | 1.197–8.035 | 0.020 |  | 2.804 | 1.066–7.374 | 0.037 | |  |
| Sex (male) | 1.118 | | 0.590–2.117 | 0.732 |  | 1.135 | 0.594–2.167 | 0.701 | |  |
| Platelet count ≥1000 × 10^9^/L | 1.441 | | 0.764–2.718 | 0.259 |  | 1.796 | 0.938–3.439 | 0.077 | |  |
| History of CRT | 2.955 | | 1.035–8.433 | 0.043 |  | 2.798 | 0.956–8.193 | 0.061 | |  |
| Anagrelide starting dose >0.5 mg/day | 1.659 | | 0.683–4.032 | 0.264 |  | 1.645 | 0.672–4.027 | 0.276 | |  |

CRT: cytoreductive therapy; ET: essential thrombocythemia; NA: not applicable.
